# Poor-Law Guardians and the Feeble-Minded

**Published:** 1914-02-28

**Authors:** 


### POOR-LAW GUARDIANS AND THE FEEBLE-MINDED.

During the discussion on "The Poor Law and the Care and Training of Mentally Deficient Persons," at the recent Central Poor-Law Conference, Mr. Curtis, the Clerk to the Birmingham Union, who read the opening paper, and several of the subsequent speakers, commented regretfully upon the spirit of antipathy to Boards of Guardians evinced by all the Parliamentary parties during the discussions on the Mental Deficiency Act of 1913. Regret was also expressed that no powers to administer the Act had been entrusted to the Guardians. The reasons for this hostility to the Guardians on the part of Parliament are obvious to anyone acquainted with the history of the Poor Law. Speaking generally, Boards of Guardians have never endeavoured to carry out in a full and statesmanlike manner the powers they already possess for the relief of the sick poor, whether the sickness was mental or bodily in character. Judged by the records of history, the policy of most Guardians has been, and still is, to offer the minimum provision possible without outraging public opinion. The great reforms in Poor-Law work have been forced on the Guardians from without, and have not been originated by them, as should have been the case.

It was the indignation of the public, roused by the scandals revealed by the *Lancet* Commission in 1865 and 1866, which forced upon the Poor-Law Board the reform of the London workhouse infirmaries. In the provinces it was not until the investigations undertaken by the *British Medical Journal* in 1894 and 1895 showed that conditions almost as bad still existed there that reforms began to be generally adopted. Finally, it needed the investigations of the Royal Commission of 1905 to 1909 to stimulate the mass of Guardians to initiate the improvements which are now slowly taking place. How difficult it is to induce Boards of Guardians to realise their responsibilities and to take action to meet them was shown by Mr. Curtis in his paper.

In 1901 the Central Poor-Law Conference of Boards of Guardians in England and Wales unanimously adopted a resolution that it was desirable that the younger imbeciles, the epileptics, and the feeble-minded should be provided for by Boards of Guardians in special institutions, and that Boards of Guardians should combine for the establishment of such institutions. With very few exceptions, however, nothing was done, and in 1908 the Royal Commission on the Care and Control of the Feeble-Minded reported against reliance on the combination of Unions to supply the institutions necessary, unless such combinations were made compulsory. Even under the stimulus of this report and of the Mental Deficiency Act only, forty-four Unions out of over 600 have actually combined for this purpose, and seventy-nine others have prepared schemes which are now awaiting sanction.

The treatment of sane epileptics affords a further illustration of the failure of Guardians to provide accommodation for those for whom it is their duty to provide. With the notable exceptions of Birmingham, Chorlton, Manchester, and a few other Unions the only provision made by Guardians for sane epileptics is in the ordinary workhouse and workhouse infirmary, accommodation which is totally unsuited for the proper treatment of such cases. Even in London, where the number of epileptics chargeable to the Guardians would warrant the establishment of two or three epileptic colonies, completely equipped for the trial of the various forms of treatment known to be beneficial, and with provision for research work, the only accommodation available, outside the workhouses and infirmaries, consists of a few wards at Belmont and Greenwich workhouses, altogether inadequately equipped and staffed.

Quite apart from the failure of the Guardians in these special cases, the difficulty that has been experienced in the past, and that still exists, in inducing them to provide adequately for the sick justifies Parliament and the public in distrusting them, and in refusing to endow them with new powers. Before this distrust can be eradicated the attitude of the Guardians in this respect must undergo a complete reversal.

